# Dentists’ perception of health-risk associated with cigarettes and alternative tobacco products: A descriptive study

**DOI:** 10.3126/nje.v12i4.48488

**Published:** 2022-12-31

**Authors:** Danavanthi S Bangera, Mohamed Tahir Takana, Aji Gopakumar, Jayakumary Muttappallymyalil

**Affiliations:** 1 Thumbay Dental Hospital, Ajman, United Arab Emirates; 2 Al Madina Primary Health Care Centre, Ajman, United Arab Emirates; 3 Data & Statistics Department, Emirates Health Services, Dubai, United Arab Emirates; 4 College of Medicine, Gulf Medical University, Ajman, United Arab Emirates

**Keywords:** Dentists’ perception, Tobacco smoking, Health risk, Cigarettes and alternative tobacco products, Tobacco cessation advice

## Abstract

Cigarettes and alternative tobacco products cause various oral health issues ranging from minor tooth decay/gum-diseases to oral cancer. According to the Centre for Disease Control (CDC), over 40% of adult cigarette smokers have untreated tooth decay which later leads to severe oral health problems. The present study intended to assess the impact of dentists’ perceived risk of smoking tobacco products on their attitude and practice toward tobacco cessation advice. It was a cross-sectional study to collect data on the perception of dentists who are smokers, regarding smoking-related health risks. Dentists’ attitude was measured using a questionnaire with a 5-point rating scale and a practice list of items with a 3-point rating scale. Descriptive/inferential techniques were applied, and a significance level was fixed at 5%. Among the 31 tobacco users, 80.6% of dentists perceived severe risk with cigarette use as compared to alternative tobacco products (71%). Positive attitude and good practice were observed among those who perceived severe risk; however, no statistical significance was observed (p-value >0.05). Young dentists, males, Arab nationals, highly educated and specialists had a positive attitude toward smoking cessation activities. Among the smokers, dentists recognize their role and responsibility very seriously in building a smoke-free community, but their risk perception and good attitude did not positively reflect on their clinical practice in smoking cessation programs. Though the dentists had a better perception and attitude towards smoking cessation, their clinical practice in offering advising sessions is inversely related to their perception/attitude.

## Background

Smoking is a known risk factor for various diseases and disabilities, with more than 7 million deaths reported per year, worldwide. If the current smoking pattern does not change, tobacco-related diseases may increase to 8 million death per year globally. Cigarettes and other alternative tobacco products cause various oral health issues ranging from minor periodontal decay or gum diseases to oral cancer. According to the Centre for Disease Control (CDC), over 40% of adult cigarette smokers have reported untreated tooth decay which later leads to severe oral health problems. Consumption of tobacco is associated with various diseases such as cancer, lung diseases, diabetes, stroke, and other dysfunctions and is one of the leading causes of preventable death. Among various tobacco products, cigarettes are the most common form of tobacco worldwide. The pattern of tobacco use in the United Arab Emirates (UAE) shows frequent use of other tobacco products especially Shisha and Dokha in the eastern Mediterranean region. Among the deaths-related with tobacco consumption, the majority are associated with first-hand smoke and 1.2 million deaths are due to second-hand smoke. In this context, healthcare providers have a significant role in tobacco cessation programs [1-8].

Notably, dental professionals should have an active role in smoking cessation activities, worldwide. However, studies have reported poor coordination of dentists and tobacco cessation services [9]. The provision of counseling to smokers as part of dental practice and its implementation in common clinical settings is inadequate [10]. The literature recommends an active intervention of dental professionals is an effective strategy to prevent tobacco initiation among non-smokers and in reducing smoking habits in users. Therefore, clarity in dentists’ perception about providing tobacco cessation advice is vital, as there are certain barriers such as financial issues, lack of knowledge and expertise in smoking cessation services, and time constraints. Personal smoking habit is another major issue among dentists. Dental professionals who use to smoke are less likely to initiate tobacco cessation campaigns in their clinics [11]. Therefore, the current study took an attempt to reveal the perception of dentists in providing tobacco cessation services and counseling sessions in hospital settings. Since their perception has a significant role in forming a positive attitude towards quitting tobacco and support the smokers in stopping various tobacco products. It is believed that dentists’ perception will have an effect in conducting tobacco-related awareness programs and consistently keep advising sessions for smokers as part of their clinical practice. The present study intended to assess the impact of dentists’ perceived risk of smoking tobacco products on their attitude and practice toward tobacco cessation advice.

## Methods

This article is part of the research conducted to assess the role of dentists in tobacco cessation advice and to find their attitude and practice. It was a cross-sectional study to include gender and national dentists practicing in the Northern Emirates, UAE who were willing to participate were part of the study. Academicians were excluded from the research.

The number of subjects participated in the study were two hundred fifty, but the current article mainly focused on dentists who used to smoke. Non-smokers were expected to rate high-risk with any tobacco products and those participants may not be tried smoking for the probable high health risk. Therefore, the researchers were interested to know the perceived risk of dentists who are smokers. The perception, attitude, and practice of 31 dentists were comprised with regard to the offering of smoking cessation activities as part of their dental practice. Participation was voluntary and withdrawal from the study was allowed at any point of time. The questionnaire included open and closed-ended questions under the main domains such as socio-demographic characteristics, type of practice, history and current habits of smoking, perceived risk in using various tobacco products, attitude, and practice of dentists toward tobacco cessation programs. Dentists’ attitude toward the provision of tobacco cessation advice was measured on a 5-point Likert scale with a total of 22 statements under various domains. Their practice was also quantified on 22 items with 3-point Likert scale options. The total score was calculated for attitude and practice considering the possible minimum and maximum score along with the average score. Attitude and practice domains were categorized to the dentists who obtained below and above the average score. The data was collected and recorded in an Excel spreadsheet and analysis was performed using SPSS version 22. Results are presented in tabular and graphical forms to show frequency and percentages. Fisher’s exact-test was used to test the significance for the association between dependent and independent variables. The level of significance was set at p value ≤0.05.

## Results

Data included information collected from 250 dentists. Descriptive information on dentists’ perception and its impact on their attitude and clinical practice in relation to the tobacco cessation program is presented under different domains. These details are described among the dentists who were current smokers and those who practiced in the Northern Emirates of UAE (31, 12.4%).

Among the smokers, 94% of the participants were males and 64.5% were general practitioners. The age of the participants ranged between 28 to 60 years, and 52% were of age 40 years and above. The mean age of the dentists was 39.6±7.6. Thirty six percent of the dentists had more than 15 years of experience, and only 7% reported to have more than 15 years of experience in UAE. 61% had education up to graduate level and 39% were postgraduates; 36% were specialists and the remaining dentists were general practitioners. Age (mean ± SD, in years) was 39.6±7.6The results are detailed in **table 1**.

**Table 2** shows the perceptions of dentists who were smokers regarding the level of risk involved in smoking different types of tobacco products as they may have various reasons for the rated perceived risk. Based on the perception of dentists, it is observed that severe risk was associated with cigarette use. Compared to any other type of alternative tobacco products, a high risk was perceived for cigarettes (81%), the most common form of tobacco. Chewing tobacco (77%), sniffing tobacco (71%), Shisha (65%), and cigars (65%) were the other forms of tobacco rated with severe risk.

Among the smoking dentists, low-risk (48%) was perceived for smoking bidi as compared to any other tobacco products that are commonly used in UAE. 81% of dentists had a perception that the use of cigarettes leads to severe health risks, whereas only 71% rated severe health risks with alternative tobacco products.

**Table 2** also shows the association of dentists’ demographic factors with their perceived risk in smoking Cigarettes. It was found that the doctors’ risk perception does not vary according to the demographic characteristics (p-value >0.05). Compared to females, male doctors (83%) perceive severe health risks from cigarette smoking, notably, there were only 2 dentists females. In other comparisons, severe health risk was perceived by South East Asians (91%) and dentists who are highly educated (92%). The level of risk was rated as the same among general practitioners and specialists. Experienced dentists and less experienced participants perceived the same rate of risk for cigarettes and alternative tobacco products.

For smokers of alternate tobacco products (**Table 3**), the same kind of trend was observed except for some indicators. Doctors from Southeast Asia perceived severe health-related risks of using any type of tobacco product. The severe health risk was perceived more by the doctors who are highly educated and with specialization. The perceived level of health risk of using cigarettes slightly increases with increasing years of practice and number of patients treated. However, for the alternative tobacco products, it was found the other way. Doctors who have more years of experience in the UAE perceived severe risk for using cigarettes in comparison with any type of tobacco product. Low risk was anticipated by the experienced dentists and those who have more patients visits per week. This perception may be the main reason for the higher use of alternative tobacco products in the northern emirates of UAE. The majority perceived less risk in alternative tobacco products compared to cigarettes. But the observed pattern was not statistically significant in the population, though the sample selected depicted a particular trend in the dentist’s perception of associated health risk.

**Table 4** shows the risk level perceived by the dentists in smoking according to their cultural diversity and age. High health risks from cigarettes are perceived by dentists in the age category of >40 years (88%), but young dentists (80%) rated severe health issues from alternative tobacco products. A good attitude towards smoking cessation was observed among young doctors compared to aged dentists. Both young and aged dentists were noticed with the poor practice of offering advising cessations on quitting tobacco. Compared to non-Arabs (78%), 85% of Arab nationals perceived severe risk with cigarette use. Likewise, 67% of dentists rated low risk and 77% perceived high risk with regard to the use of alternative tobacco products. Dentists who currently smoke and from Arab countries had a perception of severe risk with smoking any type of tobacco product. However, the observed pattern was not statistically significant. A good attitude score was observed among 77% of Arabs, but only 31% of Arabs had a good practice score with respect to tobacco cessation. Results indicate that Arabs were more efficient and responsible in providing awareness on quitting tobacco for a healthy society and recognizing their role in building a smoke-free community, but their score in clinical practice of giving tobacco cessation advice was found poor.

Scientifically, one’s perception has a significant impact on their attitude and practice in providing smoking cessation advice. Good attitude and practice scores were found among those who perceived severe risk for tobacco users (p>0.05, **Table 5**). Though the dentists had a better perception and attitude towards advice for stopping the use of tobacco products, their performance in clinical advising for smoking cessation needs to be improved further.

## Discussion

The current paper was focused on the perception, attitude, and practice of dentists who were smokers toward smoking cessation programs. A study was essential in identifying dentists’ perceptions regarding the risk involved in the use of various tobacco products and its impact on their clinical practice toward tobacco quitting programs. There are few studies in the literature that investigated patients’ perceptions about the role of dentists in smoking cessation. An earlier study from India reported a positive perception among patients, about the role of dentists in tobacco cessation programs and whether patients were willing to quit tobacco, if suggested by the dentist [12]. The current paper is part of the main research which also established the same facts. A parent study identified good attitudes among 88% of dentists, but the good practice of providing tobacco cessation advice to their patients was followed by only 37% of dentists. In a study among dental students in UAE regarding factors leading to the habit of Shisha smoking, participants perceived that shisha smoking causes serious health-related problems, and the study recommended incorporation of intervention programs in the university curricula [13].

An opinion paper discusses implications for the dental team in giving awareness about the health risks of waterpipe tobacco smoking and stated dental teams are now increasingly being encouraged to promote health through community events and other outreach activities. The study reveals dental team could play a significant role in creating awareness of the health risks of shisha in educational institutions and among adolescent groups [14]. Research also reveal that there is emerging evidence of health risks with smoking shisha. It was found that shisha use was significantly associated with lung cancer, cardiovascular diseases, and increased risk of infection, especially in the Middle Eastern region [15]. Dental professionals’ attitude towards factors that may assist in smoking cessation identified general practitioners, followed by family members as the primary providers of counseling and the most important factor for providing assistance with smoking cessation. 43% of Dental practitioners and 52% of dental students responded positively in providing smoking cessation assistance [16].

Another study that investigated dentists’ attitudes, barriers, and promotion practices regarding smoking cessation observed that 81% of dentists perceive to have a vital role in smoking cessation and 54% of dentists practice advising patients to quit smoking [17]. An earlier review searching health effects of waterpipe smoking (hookah, shisha, goza, narghileh, arghileh and hubble-bubble) concluded water pipe smoking-related health risks to increased heart rate, blood pressure, impaired pulmonary function, Chronic bronchitis, coronary artery disease etc. The review contradicts widely held misconception of less risk associated with waterpipe smoking compared to other tobacco products [18]. Dental practitioners working in the public and private sectors in Al-Madinah Al-Munawarah, of Saudi Arabia reported perceptions of dental practitioners regarding health risks involved in the use of various tobacco products. 94% of the dentists perceived waterpipe smoking is highly harmful to health. 50% of the participants believed that waterpipe smoking is more harmful than cigarettes, and 39% believed cigarettes are more harmful than waterpipe smoking [19]. The current study also revealed the perception of the majority of the dentists (81%) as cigarettes are more harmful than any other alternative tobacco products. Evidence shows that cigarette smoking has declined in the last few years, especially in the developed countries, and increased waterpipe smoking. But no evidence is available for less serious effects of smoking water pipe. The survey shows that 95% of respondents do not perceive waterpipe smoking as less addictive compared to cigarette smoking, and hence lesser health risk was perceived [20]. Although, the dentists perceived various levels of health risk involved in different types of tobacco products, lack of training on smoking cessation (13%). The smoking habit of the dental team (57%) and patients’ resistance to quitting tobacco (50%) are the major obstacles to deliver proper tobacco cessation advice [21].

Overall, smoking dentists from Arab countries had reported their perception of severe health risks with any type of tobacco product. Young dentists, males, Arab nationals, highly educated and specialists had a positive attitude toward smoking cessation activities. Though their clinical practice in the work settings towards tobacco cessation advice was found poor.

## Conclusion

The majority of the dentists perceived severe risk associated with cigarette use as compared to alternative tobacco products. Doctors who have more years of experience in the UAE and those who are highly educated and with specialization perceived severe risk with any type of tobacco products. Good attitude and best practice were observed among the doctors who perceived severe risk for tobacco users, however, no statistical significance was observed for the association between doctors’ perception of attitude and practice towards tobacco cessation. Young dentists, males, Arab nationals, highly educated, and specialists showed good attitude toward smoking cessation activities. It is an indication of dentists’ awareness regarding their vital role in building a smoke-free community. However, the clinical practice for offering advising sessions for cessation was inversely related to the perception and attitude. Therefore, it is recommended to invest more time and effort in smoking cessation activities in the workplace and clinical settings, as part of regular dental practice.

## Figures and Tables

**Table 1:** Dentists’ demographic characteristics

Demographic characters of smokers (n=31)		Frequency	Percent
**Age**	<40 years	15	48.4
	>= 40 years	16	51.6
**Gender**	Male	29	93.5
	Female	2	6.5
**Ethnic**	Arabs	13	41.9
	others	18	58.1
**Education**	UG	19	61.3
	PG & higher	12	38.7
**Specialization**	General Practitioners	20	64.5
	Specialties	11	35.5
**Total duration of practice**	<=15years	20	64.5
	> 15years	11	35.5
**UAE experience**	<=15years	29	93.5
	> 15years	2	6.5

**Table 2:** Dentist’s perceived risk in smoking Cigarettes and other tobacco products (n=31)

Perceived risk level on health with the use of various tobacco products (n=31)		Severe-risk		Low-risk	
**Tobacco products**	Cigarettes	25	80.6%	6	19.4%
	Shisha/water pipes	20	64.5%	11	35.5%
	Midwakh	17	54.8%	14	45.2%
	Chewing tobacco	24	77.4%	7	22.6%
	Sniffing tobacco	22	71.0%	9	29.0%
	Smoking bidi	16	51.6%	15	48.4%
	Smoking cigar	20	64.5%	11	35.5%

Dentist’s perceived risk in smoking cigarettes according to their demographic factors (n=31)		Severe-risk		Low-risk	
**Gender**	Male	24	82.8%	5	17.2%
	Female	1	50.0%	1	50.0%
**Nationality**	Eastern Mediterranean	15	75.0%	5	25.0%
	South East Asia	10	90.9%	1	9.1%
**Qualification**	Graduate	14	73.7%	5	26.3%
	Postgraduate & higher	11	91.7%	1	8.3%
**Job status**	General Practitioner	16	80.0%	4	20.0%
	Specialities	9	81.8%	2	18.2%
**Total years of practice**	<15 years	16	80.0%	4	20.0%
	≥15years	9	81.8%	2	18.2%
**years of practice in UAE**	< 5years	6	75.0%	2	25.0%
	5-15 years	17	81.0%	4	19.0%
	> 15years	2	100.0%	0	0.0%
**No. of patients**	<60	11	73.3%	4	26.7%
**visits / week**	60-100	11	91.7%	1	8.3%
	>100	2	100.0%	0	0.0%

**Table 3:** Dentist’s perceived risk in smoking Alternative tobacco products according to their demographic factors (n=31)

Socio-demographic factors		Severe-risk		Low-risk	
**Gender**	Male	21	72.4%	8	27.6%
	Female	1	50.0%	1	50.0%
**Nationality**	Eastern Mediterranean	14	70.0%	6	30.0%
	South East Asia	8	72.7%	3	27.3%
**Qualification**	Graduate	12	63.2%	7	36.8%
	Postgraduate & higher	10	83.3%	2	16.7%
**Job status**	General Practitioner	14	70.0%	6	30.0%
	Specialities	8	72.7%	3	27.3%
**Total years of practice**	<15 years	15	75.0%	5	25.0%
	≥15years	7	63.6%	4	36.4%
**years of practice in UAE**	< 5years	7	87.5%	1	12.5%
	5-15 years	14	66.7%	7	33.3%
	> 15years	1	50.0%	1	50.0%
**No. of patients**	<60	11	73.3%	4	26.7%
**visits / week**	60-100	8	66.7%	4	33.3%
	>100	1	50.0%	1	50.0%

**Table 4:** Dentist’s diversity on the perceived risk associated with cigarettes and alternative tobacco products use (n=31)

Dentist’s diversity on perceived risk associated with cigarettes use		Severe Risk in cigarettes use		Low Risk in cigarettes use	
**Nationality**	Arabs	11	84.6%	2	15.4%
	Other nationals	14	77.8%	4	22.2%
**Age**	<40 years	11	73.3%	4	26.7%
	>= 40 years	14	87.5%	2	12.5%

Dentist’s diversity on perceived risk associated with ATP use		Severe Risk in ATP use		Low Risk in ATP use	
**Nationality**	Arabs	10	76.9%	3	23.1%
	Other nationals	12	66.7%	6	33.3%
**Age**	<40 years	12	80.0%	3	20.0%
	>= 40 years	10	62.5%	6	37.5%

Dentist’s diversity on attitude towards tobacco cessation program		Poor attitude score, needs improvement		Good attitude score	
**Nationality**	Arabs	3	23.1%	10	76.9%
	Other nationals	6	33.3%	12	66.7%
**Age**	<40 years	4	26.7%	11	73.3%
	>= 40 years	5	31.3%	11	68.8%

Dentist’s diversity on dental practice towards tobacco cessation sessions		Poor practice score, needs improvement		Good practice score	
**Nationality**	Arabs	9	69.2%	4	30.8%
	Other nationals	14	77.8%	4	22.2%
**Age**	<40 years	11	73.3%	4	26.7%
	>= 40 years	12	75.0%	4	25.0%
